# Habituation as an adaptive shift in response strategy mediated by neuropeptides

**DOI:** 10.1038/s41539-017-0011-8

**Published:** 2017-08-18

**Authors:** Evan L. Ardiel, Alex J. Yu, Andrew C. Giles, Catharine H. Rankin

**Affiliations:** 1 Djavad Mowafaghian Centre for Brain Health, University of British Columbia, 2215 Wesbrook Mall, Vancouver, BC Canada V6T 2B5; 2 Department of Psychology, University of British Columbia, 2136 West Mall, Vancouver, BC Canada V6T 1Z4

## Abstract

Habituation is a non-associative form of learning characterized by a decremented response to repeated stimulation. It is typically framed as a process of selective attention, allowing animals to ignore irrelevant stimuli in order to free up limited cognitive resources. However, habituation can also occur to threatening and toxic stimuli, suggesting that habituation may serve other functions. Here we took advantage of a high-throughput *Caenorhabditis elegans* learning assay to investigate habituation to noxious stimuli. Using real-time computer vision software for automated behavioral tracking and optogenetics for controlled activation of a polymodal nociceptor, ASH, we found that neuropeptides mediated habituation and performed an RNAi screen to identify candidate receptors. Through subsequent mutant analysis and cell-type-specific gene expression, we found that pigment-dispersing factor (PDF) neuropeptides function redundantly to promote habituation via PDFR-1-mediated cAMP signaling in both neurons and muscles. Behavioral analysis during learning acquisition suggests that response habituation and sensitization of locomotion are parts of a shifting behavioral strategy orchestrated by pigment dispersing factor signaling to promote dispersal away from repeated aversive stimuli.

## Habituation is more than learning to ignore

The long held notion that habituation is simply learning to ignore a repeated stimulus has been challenged by this work showing that habituation reflects a shift in response strategy to escape a repeated aversive stimulus. The laboratory of Dr. Catharine Rankin at the University of British Columbia used a microscopic roundworm (*C. elegans*) to investigate habituation to aversive stimuli in an attempt to understand why an animal would decrease responding to something potentially dangerous. Using computer vision software for detailed behavioral analyses, they found that for *C. elegans*, habituation is part of a behavioral strategy to disperse to safety away from the site of stimulation. Through a genetic analysis they identified a key signaling system underlying this process. This work reframes the purpose of habituation and begins to reveal underlying cellular and circuit processes

## Introduction

Habituation is a form of non-associative learning characterized by a decremented response to repeated sensory input. It has been documented across phylogeny and is often considered a cognitive “building-block”.^1^ Consistent with this role, deficits in habituation are associated with a variety of neuropsychiatric disorders, including autism and schizophrenia.^2^ Habituation is typically framed as a process to free up limited neuronal resources by allowing organisms to ignore irrelevant stimuli. In conflict with this characterization are reports of habituation to stimuli that are potentially lethal. As an example, the nematode *Caenorhabditis elegans* habituates to repeated activation of a pair of polymodal nociceptor neurons.^3, 4^ These neurons, named ASH, receive input at the worm’s nose and elicit a rapid reversal response to chemical repellents (many of them toxic), osmotic pressure (potentially lethal), and physical contact.^5–8^ This behavior is of particular interest in the context of habituation, both because of the diversity of the cues detected by ASH and because of the potential lethal consequences of “ignoring” such stimuli.

Most learning studies tend to be response-centric, that is focusing solely on measuring a single-response metric without considering changes in other components of behavior. This may offer only an incomplete picture of the effect that the stimulus has on overall behaviors. In this study, we consider not only the plasticity of the components of the ASH reversal response, but also how this changing response relates to the ongoing behavior of the animal. Analysis of changes in multiple behavioral components of the ASH-driven response suggested a coordinated alteration in strategy to facilitate dispersal away from potentially lethal stimuli. Pigment-dispersing factor (PDF) signaling was essential for this process and tissue-specific expression experiments demonstrated that the PDF receptor, PDFR-1, inhibited reversal responses and facilitated dispersal by promoting cAMP production in neurons and muscles. Our experiments demonstrate that *C. elegans* shift behavioral strategies from a backwards escape response in order to evade an immediate threat to a rapid forward acceleration attempting to evacuate away from the locus of the stimulation. This study provides a strong hypothesis and mechanism to explain why organisms habituate to aversive stimuli.

## Results

### Responding to repeated ASH photoactivation requires GLR-1

**Fig. 1:**
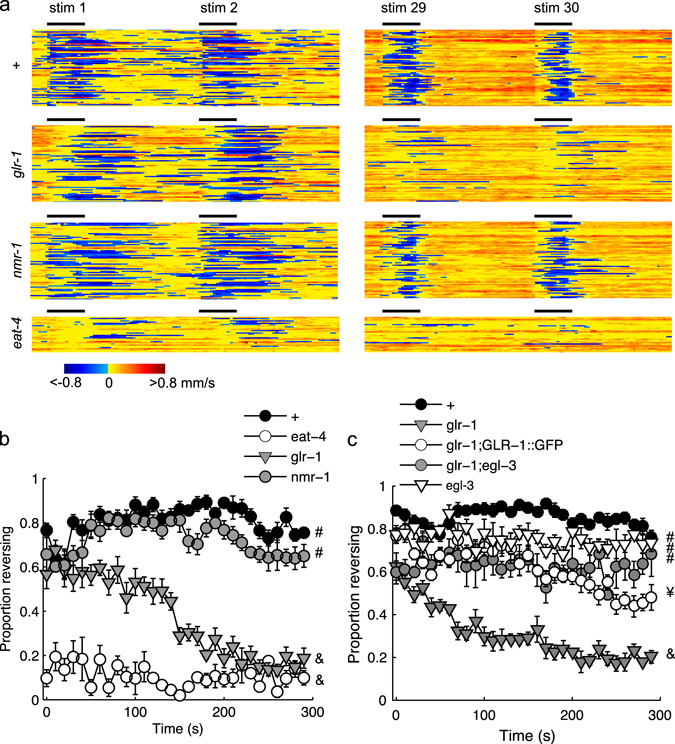
Habituation of glutamate transmission mutants reveals a *glr-1* mutant phenotype. **a** Representative raster plots depicting the behavioral state at the beginning (*left*) and end (*right*) of training. Pixels are color coded for speed with negative values corresponding to backward locomotion. *Black bars* indicate 2 s of whole-plate illumination with *blue light* at 250 μW/mm^2^. **b**, **c** Proportion of the population reversing to each of thirty 2 s light pulses administered at 0.1 Hz. **b** The *glr-1*-independent response did not persist across the assay, **c** but could be rescued with a GLR-1 expressing transgene (GLR-1::GFP) or suppressed by loss of *egl-3*. Mean ± SEM. ‘#’, ‘¥’, and ‘&’ denote groups that are statistically different based on the likelihood of responding to the final stimulus. *N* = 6 plates/strain

We previously established a high-throughput habituation assay of the ASH avoidance circuit, such that repeated ASH photoactivation results in longer response latency and shorter duration reversals, with very little decrement in the probability of responding.^3^ In investigating molecular components mediating this behavioral plasticity, we evaluated the loss-of-function phenotypes of several glutamate transmission mutants: *glr-1* (lacking an AMPA receptor subunit), *nmr-1* (lacking an NMDA receptor subunit), and *eat-4* (lacking a vesicular glutamate transporter). While control animals, and those lacking NMR-1, maintained a high probability of responding to repeated 2 s light pulses at 0.1 Hz, loss of EAT-4 eliminated reversal responses, and loss of *glr-1* led to animals that responded initially, but were unresponsive by the end of the trial (Fig. 1a, b). The phenotype of the *glr-1* mutant could be at least partially rescued with a GLR-1::GFP transgene (Fig. 1c), confirming the *glr-1* mutation as the causative allele. Note that the duration and latency habituation metrics are difficult to interpret for the *glr-1* mutant because the small number of animals responding after 30 stimuli greatly increased variability of these measures.

### Habituation is mediated by neuropeptides

Previous studies demonstrated that the *glr-1* phenotypes associated with naive ASH-mediated responses could be at least partially suppressed by disrupting neuropeptide synthesis, demonstrating that inhibitory peptides modulate this avoidance circuit.^9, 10^ To test whether neuropeptides were involved in habituation of ASH-mediated responses, we disrupted neuropeptide synthesis with a mutant allele of EGL-3, a proprotein convertase required for neuropeptide synthesis.^11, 12^ Indeed the habituation phenotype of the *glr-1* mutant was suppressed by disrupting neuropeptide processing, as a *glr-1;egl-3* double mutant was more responsive at the end of the assay than the *glr-1* single mutant (Fig. 1c). Thus GLR-1 is essential for sustained reversal responses to repeated ASH activation only in the presence of neuropeptides. Like control animals, the *egl-3* single mutant maintained a high probability of responding across the trial (Supplementary Fig. S1A), however unlike controls the *egl-3* mutant did not display response latency habituation (Supplementary Fig. S1B). Loss of *egl-3* also affected the shape of the reversal duration habituation curve, but this appeared to be primarily caused by short initial responses that did not change in duration across the 30 stimuli (Supplementary Fig. S1C). To confirm the role of neuropeptides in response latency habituation, we tested a mutant lacking EGL-21, a carboxypeptidase required for the synthesis of neuropeptides.^13, 14^ As with EGL-3, loss of EGL-21 disrupted habituation of response latency and duration (Supplementary Fig. S1B), suggesting that peptidergic signaling promotes habituation of ASH-mediated reversals.

### GPCR RNAi suppressor screen

**Fig. 2:**
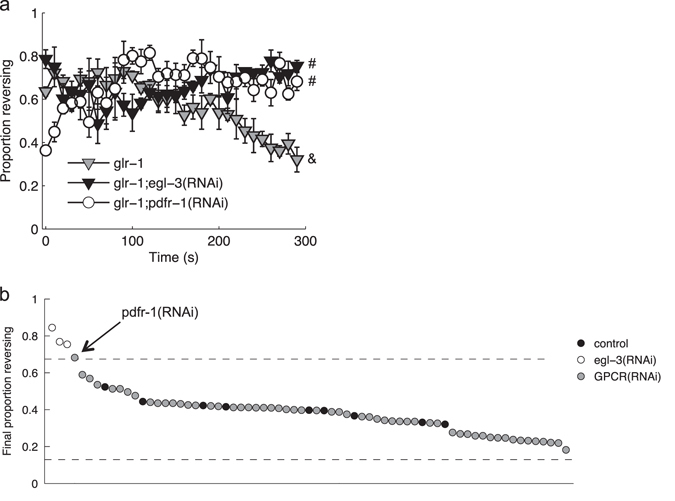
GPCR RNAi screen. **a** Proportion of the population reversing to each stimulus for animals fed RNAi clones that increased the probability of a reversal to the final stimulus. Mean ±SEM. ‘#’ and ‘&’ denote significantly different groups based on the likelihood of responding to the final stimulus. **b** Proportion of animals reversing to the final stimulus for populations fed RNAi clones to knockdown *egl-3* or one of 57 GPCRs. Knockdown was done in a background sensitized to neuronal RNAi by feeding: *glr-1*; *lin-15b*; *sid-1*; *unc-11*9p::*sid-1*. Each circle is the mean of three plates, with multiple replicates for the control and *egl-3* targeting vector. *Dashed lines* mark upper and lower critical values (values corresponding to |*z*-score| > 4.46; *P* < (0.05/57 =) 0.0008)

The *C. elegans* genome has 119 neuropeptide precursor genes that can be processed into over 250 peptides that signal through at least 128 neuropeptide G-protein-coupled receptors.^15, 16^ To identify the neuropeptide signal or signals responsible for *egl-3* suppression of *glr-1*, we used systemic RNAi to knockdown known and predicted neuropeptide receptors in a *glr-1* mutant background. The nervous system of *C. elegans* is generally refractory to RNAi by feeding, but can be sensitized by neuron-specific expression of the dsRNA channel, SID-1, in a *lin-15*b mutant background.^17^ While the previous experiments reported here used a transgene dependent on FLP Recombinase to specifically target ChR2 to ASH,^18^ a more responsive strain with a highly expressing *sra-6*p::ChR2 transgene was used for the RNAi screen. The *sra-6* promoter expresses strongly in ASH and more weakly in a pair of sensory neurons (ASI) and interneurons (PVQ).^19^ The increased sensitivity of this strain allowed for the testing of more animals, as a larger surface area could be illuminated at irradiance sufficient for robust responding. We first confirmed that RNAi knockdown of *egl-3* promoted responding in this genetic background: as predicted, a *glr-1* mutant sensitized for neuronal systemic RNAi was more likely to reverse after repeated photoactivation of *sra-6*-expressing cells if fed bacteria expressing double-stranded RNA complementary to *egl-3* (Fig. 2a). Using the RNAi by feeding approach, we evaluated the reduction of function phenotype for known and predicted neuropeptide GPCRs (Fig. 2b; Supplementary Table S1). After correcting for multiple comparisons, only knockdown of PDFR-1 suppressed the decreased response phenotype of the *glr-1* mutant.

### PDFR-1 signaling in neurons and muscles mediates habituation

**Fig. 3:**
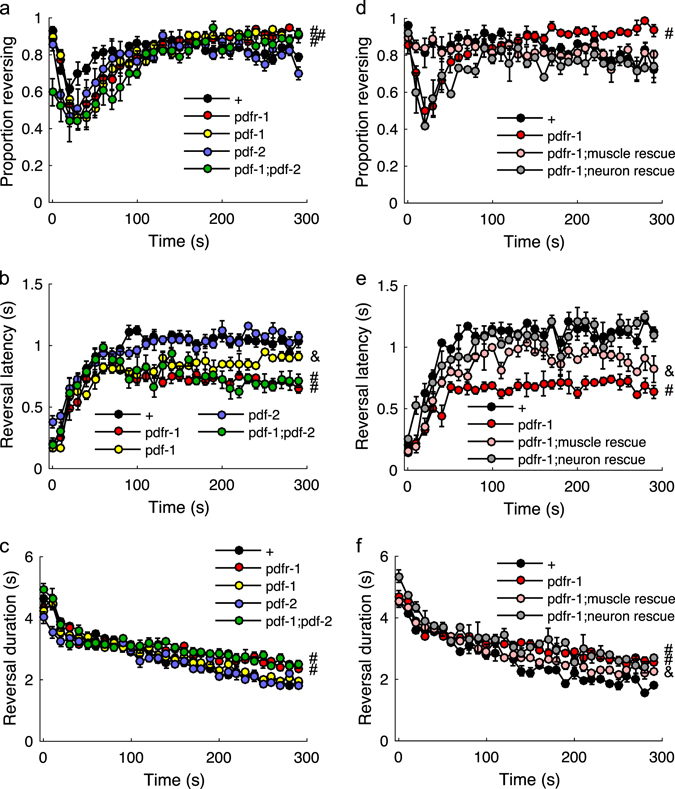
PDF signaling promotes habituation. Proportion of the population reversing (**a**), reaction time (**b**), and response duration (**c**) to each of thirty 2 s light pulses administered at 0.1 Hz. **d**–**f** Effects of restoring PDFR-1 expression to neurons or muscles. Mean ± SEM. ‘#’ denotes groups significantly different from control and ‘&’ denotes groups significantly different from both control and the *pdfr-1* mutant based on the response to the final stimulus. *N* = 2–4 plates/strain

PDFR-1 is a member of the secretin receptor family and is associated with a state of arousal in both worms and flies.^20–23^ To evaluate the RNAi result, we tested a *pdfr-1* mutant in the habituation assay. Compared to control, loss of *pdfr-1* only slightly, but significantly, increased the probability of reversing to the final stimulus of habituation training (Fig. 3a), but robustly decreased response latency (Fig. 3b) and increased response duration (Fig. 3c) in the latter half of the habituation series. PDFR-1 has three known ligands encoded by two precursor genes, *pdf-1* and *pdf-2*.^24, 25^ Loss of *pdf-1* alone had an intermediate habituation phenotype for response latency (Fig. 3b), while loss of *pdf-2* alone had no effect, and simultaneous loss of *pdf-1* and *pdf-2* recapitulated the loss of the receptor (Fig. 3a–c). The same habituation deficit for the *pdfr-1* mutant was apparent when ChR2 was targeted specifically to ASH, as opposed to all *sra-6* expressing cells (Supplementary Fig. S2).

PDFR-1 is expressed in body wall muscle cells, as well as in neurons in the head and tail.^20, 24^ In an attempt to identify in which tissue PDFR-1 functioned to promote habituation, we used an intersectional promoter rescuing strategy, in which Cre recombinase expression vectors were co-injected with inverted and floxed *pdfr-1* cDNA driven by the *pdfr-1* promoter.^21^ Full rescue was defined as scores distinct from the mutant and not significantly different from the control. Pan-neuronal expression of Cre (*tag-168* promoter) fully rescued the response probability and latency phenotypes (Fig. 3d, e); however, none of the lines tested rescued response duration habituation. Using several different promoters to target Cre to subsets of *pdfr-1*-expressing neurons, we only ever observed a partial rescue for response latency (Supplementary Table S2), suggesting PDFR-1 is functioning in a distributed network. In contrast, restoring *pdfr-1* expression in the body wall muscle (*myo-3* promoter) fully restored response probability and partially rescued the response latency (Fig. 3e) and duration phenotypes (Fig. 3f). Full rescue of response duration was not observed, even when *pdfr-1* expression was simultaneously restored to muscles and neurons (data not shown). Thus, probability, duration, and latency represent dissociable metrics mediated by PDFR-1 signaling in multiple cells types.

### Elevated cAMP in PDFR-1-positive cells promotes habituation

**Fig. 4:**
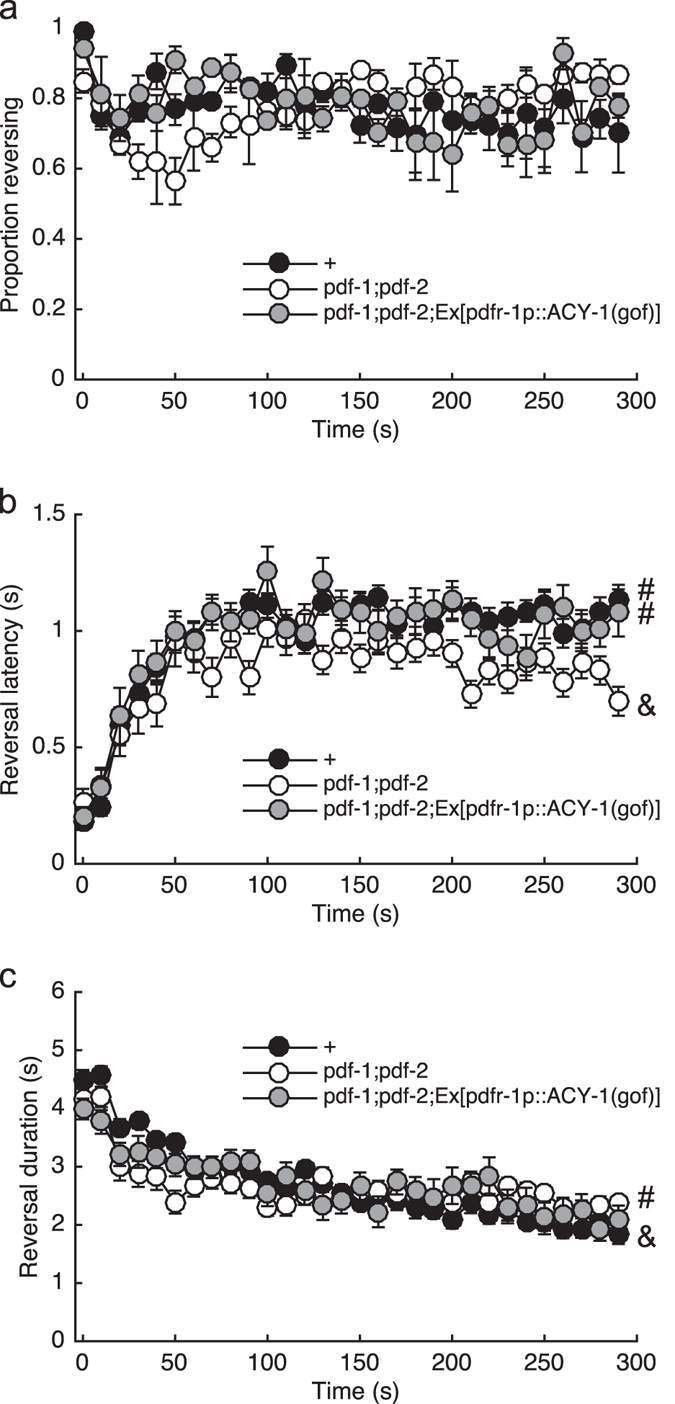
PDFR-1 signals through cAMP to promote habituation. Proportion of the population reversing (**a**), reaction time (**b**), and response duration (**c**) to each of 30 2 s light pulses administered at 0.1 Hz. Mean  ±  SEM. ‘#’ and ‘&’ denote significantly different groups based on the response to the final stimulus. *N* = 3 plates/strain

As with many secretin receptors, PDFR-1 is thought to signal through Gαs to stimulate cAMP synthesis by adenylyl cyclase. Indeed HEK239 cells expressing PDFR-1 showed a dose-dependent increase in cAMP levels with PDFR-1 activation.^24^ To test whether elevating cAMP levels in *pdfr-1* expressing cells could be used to normalize behavior in the absence of PDF neuropeptides, we used the *pdfr-1* promoter to drive expression of a constitutively active adenylyl cyclase (ACY-1(P260S)^26^) in the *pdf-1*;*pdf-2* double mutant background. Stimulating cAMP synthesis compensated for loss of the PDF ligands, as response latency habituation of the *pdf-1;pdf-2* double mutant expressing constitutively active ACY-1 was not significantly different from control (Fig. 4b). The results were less clear with the reversal duration metric, as the rescue line was intermediate and was not significantly different from either mutant or wild type (Fig. 4c). It is important to note that this chronic elevation of cAMP did not simply induce a habituated state, as initial responses were unaffected. This suggests that PDF signaling is permissive (rather than instructive) for habituation of ASH-mediated responses.

### PDF signaling promotes dispersal to repeated sensory input

Spontaneously behaving worms occupy at least two distinct behavioral states: roaming and dwelling.^27, 28^ The states are characterized by different sequences of common motor patterns, with roaming promoting dispersal through faster movement and fewer turns and reversals. PDF signaling appears to promote roaming over dwelling behavior in both males and hermaphrodites.^20, 21, 29^ This behavior has been attributed to sensory, motor, and interneurons, as well as muscle.^20–22^ We evaluated spontaneous locomotion by quantifying displacement over the 30 s window immediately preceding the habituation assay. Consistent with its previously reported propensity for a dwelling over roaming state,^21^ we observed that the *pdfr-1* mutants traveled shorter distances than control (Supplementary Fig. S3). Loss of either *pdf-1* or *pdf-2* caused a similar, but less severe phenotype that was additive in the *pdf-1;pdf-2* double mutant (Supplementary Fig. S3), supporting earlier findings that the PDF ligands function partially redundantly to promote roaming via the PDFR-1 receptor.^21, 29^

**Fig. 5:**
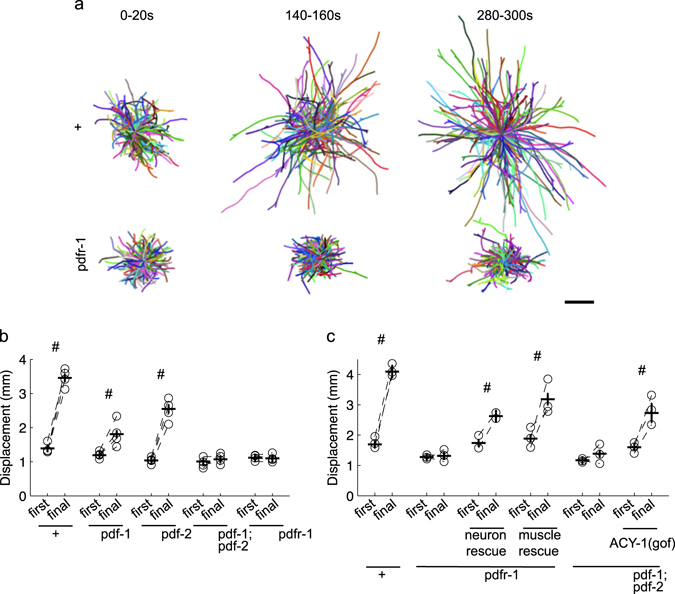
PDF signaling promotes dispersal during habituation training. **a** Representative trajectories during a 20 s interval at the beginning (0–20 s), middle (140–160 s), and end (280–300 s) of the habituation assay with thirty 2 s light pulses delivered at 0.1 Hz. Individual tracks were randomly assigned colors and set to start from the same point. *N* = 4 plates/strain. *Scale bar* = 1 mm **b** Displacement (shortest distance between the start and endpoint) over the first and final 30 s of the assay for the PDF signaling mutants. **c** Displacement over the first and final 30 s of the assay for PDF signaling mutant rescue lines. ‘#’ denotes a significant increase in displacement (one-tailed, *P* < 0.01, with Bonferroni correction for multiple comparisons) over the assay. *Circles* are plate means, *crosses* are population means  ±  SEM

Given the role of PDF signaling in habituation (Fig. 3) and spontaneous dispersal (Supplementary Fig. S3), we evaluated the influence of repeated ASH activation on locomotion during habituation by quantifying displacement over the first and final 30 s of the habituation assay. Within-strain comparisons revealed an increase in distance traveled in the final 30 s compared to the initial 30 s for the control animals that was not apparent for either the *pdfr-1* mutant or the *pdf-1;pdf-2* double mutant (Fig. 5a, b). The habituation-induced dispersal deficit of the *pdfr-1* mutant could be rescued by restoring PDFR-1 expression to neurons or muscles (with the full extent of the behavior rescued by simultaneous expression in both tissues) and the deficit of the *pdf-1*;*pdf-2* double mutant could be rescued by elevating cAMP in PDFR-1-expressing cells (Fig. 5c). The habituation-induced dispersal deficit for the *pdfr-1* mutant was also apparent using the transgenic strain in which ChR2 expression was restricted to ASH (Supplementary Fig. S2), ruling out an essential contribution from ASI and PVQ in mediating this behavioral plasticity.

**Fig. 6:**
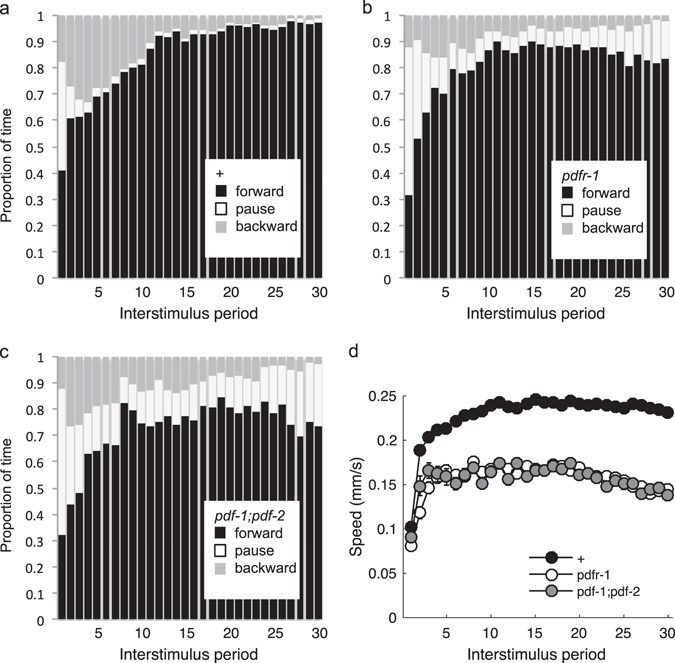
Shift in locomotion between stimuli. Proportion of the population’s time spent moving forward, backward, or not at all during the 3 s interval immediately preceding each stimulus delivered at 0.1 Hz for control (**a**) and *pdfr-1* (**b**) and *pdf-;pdf-2* (**c**) mutants. **d** Speed of worms moving forward during the same 3 s intervals. Mean ± SEM. *N* = 6 plates/strain

The shorter reversal responses associated with habituation would be predicted to facilitate dispersal in an environment with persistent ASH-sensed stimuli. However, the partial habituation phenotype of the PDF signaling mutants seemed insufficient to explain the marked difference in training-induced dispersal (Fig. 5). We therefore examined locomotion in the intervals between stimuli and observed that repeated ASH activation was associated with an increase in the probability of forward movement (Fig. 6a) and a marked increase in forward speed (Fig. 6d) in the 3 s period immediately preceding stimuli. While these behavioral changes may be necessary to increase displacement, they are not sufficient, as locomotion of the *pdfr-1* and *pdf-1;pdf-2* mutants displayed a similar pattern of plasticity, albeit with a fewer animals responding and slower speeds at the end of the assay (Fig. 6b–d). We propose that dispersal behavior is a combination of reversal response habituation and forward locomotion sensitization.

## Discussion

Using detailed behavioral analysis during learning acquisition, we identified a suite of changes associated with habituation training. Maintaining a response to repeated ASH photoactivations depended on the glutamate receptor subunit GLR-1 (Fig. 1b). The habituation phenotype of the *glr-1* mutant could be suppressed by simultaneous loss of EGL-3, a proprotein convertase required for the synthesis of neuropeptides (Fig. 1c). We performed an RNAi screen to identify the key neuropeptide receptor or receptors, ultimately identifying PDFR-1 and its ligands, encoded by *pdf-1* and *pdf-2*. PDF signaling played an important role in both habituation of ASH-mediated responses and in locomotory sensitization associated with repeated ASH activation (Figs. 3 and 5). Genetic rescue experiments suggested that elevated cAMP in *pdfr-1*-expressing neurons and muscles promoted habituation of ASH-mediated reversals and locomotory sensitization (Figs. 3d–f, 4b, and 5c).

PDF signaling is highly conserved across the protostomian evolutionary lineage. In *Drosophila*, it is a key regulator of the circadian cycle via rhythmic release to promote arousal during waking.^23, 30^ PDF receptors are distantly related to mammalian calcitonin GPCRs expressed in neurons and muscles and vasoactive intestinal peptide receptors, which appear to have conserved function regulating arousal and circadian rhythms. For *C. elegans*, Choi et al.^22^ found that secretion of PDF-1 neuropeptides is developmentally regulated and that reduced PDF-1 secretion around molting underlies the associated behavioral quiescence (worm sleep-like state). In adults, PDF peptides also appear to signal arousal, promoting roaming over dwelling behavior, with switching between these states occurring every several minutes.^21^ It is unclear what mediates the abrupt transitions during spontaneous locomotion, but our data suggest that the roaming state associated with PDF signaling can be induced by repeated sensory input. Consistent with this finding, the elevated arousal observed in worms with dysfunctional Neuropeptide Y receptor, NPR-1, is caused by enhanced sensory activity in both ASH and the body touch cells. This increased arousal can be at least partially suppressed by loss of PDFR-1.^31^

Habituation training by blue light activation of ASH appears to induce a roaming state by PDFR-1 signaling in both muscles and neurons (Fig. 5c). In addition, PDFR-1 plays a role in the kinetics of habituation of ASH response probability, duration, and latency, which can be differentially rescued by PDFR-1 signaling in neurons and muscles. Although there is a difference in baseline speed between unperturbed *pdfr-1* and control worms, there is very little difference in the initial blue light reversal response measures. Thus, the effects of *pdfr-1* on responses to repeated stimulation require *pdfr-1* mediated plasticity in both neurons and muscles. Activating the signaling cascade downstream of PDFR-1 (i.e., by elevating cAMP levels) did not induce a habituated state, suggesting PDF signaling is necessary, but not sufficient for normal habituation. Indeed other neuropeptides must be involved, as the neuropeptide synthesis mutants (*egl-3* and *egl-21*) had a more severe habituation phenotypes than the *pdfr-1* or *pdf-1;pdf-2* mutants (i.e., Supplementary Figs. S1B vs. S2B). Neuropeptides underlie a wide variety of processes and their importance in behavioral state and plasticity is an emerging trend.^32^ Insulin signaling has proven to have an especially prominent role in *C. elegans* behavioral plasticity^33–37^; however, this may be due to the use of food as an unconditioned stimulus in many associative learning paradigms. Other experience-dependent changes in behavior in *C. elegans* have also been shown to depend on neuropeptide signaling. For example, prolonged exposure to a volatile attractant initiates a neuropeptide-to-neuropeptide feedback loop causing decreased attraction^38^ and repeated mechanosensory input in a massed habituation protocol recruits dense-core vesicles to the synaptic terminals of the body touch cells, leading to increased release of a FMRFamide-related neuropeptide, FLP-20, and smaller reversal responses.^39^

Habituation is often framed as a process allowing animals to ignore irrelevant stimuli in order to free up limited cognitive resources. However, animals can also habituate to threatening or noxious stimuli, a seemingly maladaptive behavior. For example, the defensive gill and siphon withdrawal response in *Aplysia*,^40^ crab escape response to an overhead shadow,^41^ and the squid escape response to visual cues of a predator.^42^ Failure to avoid many of the stimuli detected by ASH could be fatal for *C. elegans*. Why then do reversal responses habituate? Detailed behavioral analysis of an intact freely moving animal revealed that the end point of habituation is not simply the decrement of a single-response metric, as it is so often reduced to. Our data suggest that habituation is part of a strategy to promote dispersal from a dangerous locale. Repeated stimulation actually induced a suite of behavioral changes that together defined the state of the organism. For repeated ASH photoactivation, reversal responses are largely maintained, but their duration shortens and latency increases as the population becomes more active. At the same time that the reversal responses are getting shorter, forward locomotion between responses is increasing. However, the avoidance circuit must balance this more long-term goal, with evasion of immediate threats. The maintenance of response probability and the increased response latency associated with habituation may help to strike this balance. Given the graded connection between ASH and reversal command interneurons,^43^ loss of an early immediate reversal response would allow animals to ignore less intense stimuli, such as nose touch, while maintaining sensitivity to more serious threats, such as osmotic shock.

The dual-process theory of habituation hypothesizes that each stimulus induces both local circuit habituation and organism-wide sensitization and the observed behavior reflects an integration of these two processes.^44^ In this case, coincident habituation and sensitization form an optimal escape strategy: minimize non-essential backward movement and promote forward movement. With this view, the outcome of habituation is not simply ignoring sensory input, but part of shifting the organism’s behavioral strategy to avoid dangerous stimuli. This is not inconsistent with the idea of Bolles^45^ that animals cannot afford the time to learn to avoid danger, thus they are prepared with a somewhat hierarchical series of default responses to danger known as species-specific defense responses (SSDRs). If one behavioral strategy is ineffective in decreasing the threat, the animal shifts to a different strategy. Bolles describes rodent SSDRs as freeze, flee, or fight. One can imagine that if a response to a dangerous or threatening stimulus does not decrease the threat, and the stimulus continues to occur, then the animal might shift to another strategy in an attempt to escape the stimulus. Thus habituation to a noxious stimulus might not just represent a decrease in the original response, but a shift from the original ineffective response to a different response that might decrease the threat. Although this interpretation is not expected to generalize to every habituating behavior, our approach highlights the value of detailed analysis of multiple behavioral metrics in an intact, freely moving animal, as opposed to a response-centric approach seen in the majority of learning assays.

## Methods

### Strains

Hermaphrodites were maintained on nematode growth medium (NGM) seeded with *Escherichia coli* (OP50) as described previously.^46^ Two integrated Channelrhodopsin-2 (ChR2) transgenes (gifts from William Schafer, MRC Laboratory of Molecular Biology) were used for ASH photoactivation: *ljIs105*[*sra-6*p::ChR2::YFP + *unc-122*p::GFP], which expresses ChR2 strongly in ASH and more weakly in chemosensory neuron ASI and interneuron PVQ^19^ and *ljIs114*[*gpa-13*p::FLPase + *sra-6*p::FTF::ChR2::YFP], which uses intersecting promoters and FLP recombinase to specifically target ChR2 to ASH.^18, 47^ Strains with the *ljIs114* transgene were illuminated at greater intensity and so carried a loss-of-function allele of *lite-1*, which encodes a native *C. elegans* short-wavelength light receptor.^48, 49^

AQ2235 *ljIs114 lite-1*(*ce314*) and/or AQ2026 *ljIs105* were crossed with TU3595, MT1241, CX3019, VM487, MT6308, VC671, KP1580, VC2609, FX04393, and LSC27 to generate the following strains:

VG186 *lin-15b*(*n744*); *sid-1*(*pk3321*) *ljIs105*; *uIS72*[*unc-119*p::*sid-1* + *mec-18*p::GFP + *myo-2*p::mCherry]

VG222 *egl-21*(*n611*); *lite-1*(*ce314*) *ljIs114*

VG223 *glr-1*(*ky176*); *lite-1*(*ce314*) *ljIs114*

VG227 *nmr-1*(*ak4*); *lite-1*(*ce314*) *ljIs114*

VG232 *eat-4*(*ky5*); *lite-1*(*ce314*) *ljIs114*

VG234 *egl-3*(*ok979*); *lite-1*(*ce314*) *ljIs114*

VG244 *egl-3*(*ok979*); *glr-1*(*ky176*); *lite-1*(*ce314*) *ljIs114*

VG250 *glr-1*(*ky176*); GLR-1::GFP; *lite-1*(*ce314*) *ljIs114*

VG264 *pdfr-1*(*ok3425*); *lite-1*(*ce314*) *ljIs114*

VG272 *glr-1*(*ky176*); *lin-15b*(*n744*); *sid-1*(*pk3321*) *ljIs105*; *uIs72*[*unc-119*p::*sid-1* + *mec-18*p::GFP + *myo-2*p::mCherry]

VG380 *pdf-2*(*tm4393*); *ljIs105*

VG382 *pdf-1*(*tm1996*); *ljIs105*

VG383 *pdfr-1*(*ok3425*); *ljIs105*

VG393 *pdf-2*(*tm4393*); *pdf-1*(*tm1996*); *ljIs105*

For cAMP overexpression in *pdfr-1*-positive neurons, pSF180 [*pdfr-1*p::ACY-1(P260S)-sl2-mCherry (50 ng/μl)] was injected into the gonad of the *pdf-1*;*pdf-2* double mutant VG393. For *pdfr-1* rescue experiments, pSF134 [*pdfr-1*p::inv[*pdfr-1.d*-sl2-GFP] (30–35 ng/μl)] was co-injected with one of several Cre expressing plasmids (1 ng/μl) for cell type-specific Cre-lox recombination in *pdfr-1* mutant VG383. pSF180, pSF134, and two of the seven Cre expressing plasmids (pSF11 [*tag-168*p::Cre] and pSF176 [*eat-4p*::Cre]) are described in Flavell et al.^21^ and were provided by the Bargmann lab at Rockefeller University. Also co-injected was pCFJ90 (*myo-2*p::mCherry (2 ng/μl)^50^) for use as a visible marker and pBluescript to make the total injected DNA concentration 100 ng/μl.^51^ The following strains were generated by microinjection:

VG492 *pdf-1*(tm1996); *pdf-2*(*tm4393*); *ljIs105*; *yvEx152*[*pdfr-1*p::ACY-1(P260S)-sl2-mCherry + *myo-2*p::mCherry]

VG447, VG448, VG449 *pdfr-1*(*ok3425*); *ljIs105*; *yvEx*[*pdfr-1*p::inv[*pdfr-1.d*-sl2-GFP] + *tag-168*p::Cre + *myo-2*p::mCherry]

VG485, VG486, VG487, VG488 *pdfr-1*(*ok3425*); *ljIs105*; *yvEx*[*pdfr-1*p::inv[*pdfr-1.d*-sl2-GFP] + *myo-3*p::Cre + *myo-2*p::mCherry]

VG411, VG412 *pdfr-1*(*ok3425*); *ljIs105*; *yvEx*[*pdfr-1*p::inv[*pdfr-1.d*-sl2-GFP] + *glr-1*p::Cre + *myo-2*p::mCherry]

VG434, VG438, VG441 *pdfr-1*(*ok3425*); *ljIs105*; *yvEx*[*pdfr-1*p::inv[*pdfr-1.d*-sl2-GFP] + *eat-4*p::Cre + *myo-2*p::mCherry]

VG442, VG443, VG446 *pdfr-1*(*ok3425*); *ljIs105*; *yvEx*[*pdfr-1*p::inv[*pdfr-1.d*-sl2-GFP] + *npr-1*p::Cre + *myo-2*p::mCherry]

VG481, VG82, VG483, VG484 *pdfr-1*(*ok3425*); *ljIs105*; *yvEx*[*pdfr-1*p::inv[*pdfr-1.d*-sl2-GFP] + *gcy-36*p::Cre + *myo-2*p::mCherry]

VG507, VG508, VG509, VG510 *pdfr-1*(*ok3425*); *ljIs105*; *yvEx*[*pdfr-1*p::inv[*pdfr-1.d*-sl2-GFP] + *ocr-4*p::Cre + *myo-2*p::mCherry]

VG647, VG648, VG649, VG650 *pdfr-1*(*ok3425*); *ljIs105*; *yvEx*[*pdfr-1*p::inv[*pdfr-1.d*-sl2-GFP] + *tag-168*p::Cre + *myo-3*p::Cre + *myo-2*p::mCherry].

### Plasmid construction

To generate the Cre expression vectors, promoters were subcloned into plasmid pSF11 [*tag-168*p::Cre] cut with FseI and AscI.

A 2 kb *myo-3* promoter was amplified from plasmid KP#1866 (Josh Kaplan, Harvard University) using oligos 5′-CTTAACGGCCGGCCTGTGTGTGATTGCTTTTTCACAATC-3′ and 5′-ACACTTGGCGCGCCTCTAGATGGATCTAGTGGTCGTGGG-3′.

A 2.7 kb *glr-1* promoter was amplified from plasmid pSH128 (Alexander Gottschalk, Goethe University Frankfurt) using oligos

5′-CTTAACGGCCGGCCTTTCAAGTGTCCTGTTGTC-3′ and

5′-ACACTTGGCGCGCCTGTGAATGTGTCAGATTGG-3′.

A 3.4 kb *npr-1* promoter was amplified from N2 genomic DNA using oligos

5′-CTTAACGGCCGGCCAAACGCAGTTGGCACAAAG-3′ and

5′-ACACTTGGCGCGCCTTGGCCTATGTCTGAAATTT-3′.

A 1.1 kb *gcy-36* promoter was amplified from N2 genomic DNA using oligos

5′-CTTAACGGCCGGCCATGATGTTGGTAGATGGGGTTTGG-3′ and

5′-ACACTTGGCGCGCCTGTTGGGTAGCCCTTGTTTGAATTT-3′.

A 4.8 kb *ocr-4* promoter was amplified from N2 genomic DNA using oligos

5′-CTTAACGGCCGGCCTCAAAGACCTTGGCTCCAC-3′ and

5′-ACACTTGGCGCGCCTAATACAAGTTAGATTCAGAGA-3′.

### Behavioral tracking

NGM plates were spread with 50–100 µl *E. coli* OP50 liquid culture mixed with all-trans retinal (ATR; or equal volume of ethanol vehicle) for a final plate concentration of 5 µM ATR. Plates were stored at room temperature in the dark for 24–48 h before use. For age-synchronized colonies, gravid adults were left 3–6 h to lay ~20–60 eggs before being removed from the plate. Animals were reared at 20 °C and tested as 3- or 4-day olds. Behavioral tracking occurred directly on the rearing plates, except for experiments using strains with extra-chromosomal arrays, in which case ~35 animals were picked (based on expression of the fluorescent co-injection marker) to ATR-containing food plates 24 h before testing (control worms were picked at the same time under identical conditions). Plates of a given strain were tested in a random order. Optimal sample sizes were determined from previous research.^3^

Multi-Worm Tracker software (version 1.2.0.2) was used for stimulus delivery and image acquisition.^52^ Following a 3–5 min acclimatization phase, stimuli were presented using custom-built LED rings (Luxeon Star LEDs) capable of illuminating 60 or 35 mm (diameter) Petri plates with uniform blue light (max = 70 or 250 µW/mm^2^, respectively). An orange filter prevented the blue light from entering the camera. Behavioral quantification with Choreography software (version 1.3.0_r1035^52^) used “--shadowless”, “--minimum-move-body 2”, and “--minimum-time 20” filters to restrict the analysis to animals that moved at least 2 body lengths and were tracked for at least 20 s. The MeasureReversal plugin was used to identify reversals occurring within 3 s (d*t* = 3) of the light pulse onset. Custom MatLab scripts organized and summarized Choreography output files. Each experiment was independently replicated at least twice. No blinding was necessary because the Multi-Worm Tracker scores behavior objectively.

### RNAi

Systemic RNAi was performed essentially as described.^53, 54^ RNAi plates comprised NGM agar, 1 mM IPTG, and 5 µM ATR seeded with overnight liquid culture of *E. coli* strain HT115 carrying either control plasmid L4440 or an RNAi vector targeting *egl-3* or one of 57 GPCRs. One or 2 days after seeding, VG186 adults were bleached onto the RNAi plates and the first-generation adults were tested behaviorally.

### Statistics

One-way ANOVAs and Tukey’s honestly significant difference criterion were used to compare responses between strains. For response probability, tests compared the mean from the proportion of worms responding to the final stimulus on each plate (*n* = number of plates tested). For latency, duration, and displacement metrics, data were combined across plates and comparisons were collective means from the final stimulus (latency and duration) or final 30 s of the assay (displacement; *n* = number of animals tested). Unless otherwise noted, *α* was 0.01.

### Data availability

The data sets generated during the current study are available from the corresponding author on reasonable request.

### Code availability

The code used to analyze data in the current study is available from the first author on reasonable request.

## Electronic supplementary material

Supplementary Table 1

Supplementary Table 2

Supplementary Figure 1

Supplementary Figure 2

Supplementary Figure 3

## Electronic supplementary material

**Supplementary Information** accompanies the paper on the *npj Science of Learning* website (doi:10.1038/s41539-017-0011-8).
