# Supplementary material for: Habituation as an adaptive shift in response strategy mediated by neuropeptides
Source: NPJ Sci Learn. 2017 Aug 18;2:9. doi: 10.1038/s41539-017-0011-8 (PMC6161508; doi:10.1038/s41539-017-0011-8)
Supplement: Supplementary file 1 — Supplementary Table 1 [file 41539_2017_11_MOESM1_ESM.docx]

Table S1. GPCR loss of function phenotypes reported as Z-scores.

| **Geneservice location** | **Target** | **Proportion reversing** | |
| --- | --- | --- | --- |
|  |  | **initial** | **final** |
| I-3C12 | *ckr-1* | 0.70 | -2.53 |
| I-3M14 | *nmur-4* | 0.57 | -2.55 |
| I-6K10 | *ntr-1* | 0.91 | -2.87 |
| II-1G01 | *nmur-2* | 1.50 | -1.07 |
| II-1G18 | *frpr-14* | 0.59 | -2.18 |
| II-6M20 | *npr-20* | 0.04 | -2.49 |
| II-8P03 | *dmsr-3* | 1.62 | -2.06 |
| II-9C14 | *npr-34* | 0.56 | -3.59 |
| II-9K23 | *dmsr-6* | -0.26 | -2.20 |
| III-1B07 | *ckr-2* | -0.15 | 0.49 |
| III-1H14 | *npr-15* | 3.45 | -1.08 |
| III-3D23 | *pdfr-1* | -1.96 | 4.59 |
| III-4H24 | *F59B2.13* | 0.48 | -0.44 |
| III-4N22 | *npr-29* | 0.15 | -0.10 |
| III-5C01 | *tkr-1* | -0.74 | -0.64 |
| III-5H08 | *dmsr-5* | -0.23 | -1.20 |
| IV-2L21 | *tkr-3* | -1.62 | -2.99 |
| IV-3K17 | *tkr-2* | 0.34 | -2.95 |
| IV-3L02 | *npr-35* | 0.08 | -2.79 |
| IV-3L09 | *npr-2* | 0.84 | 0.16 |
| IV-4P04 | *npr-27* | 1.04 | 0.00 |
| IV-6I19 | *npr-3* | 1.14 | -2.35 |
| IV-7G21 | *srsx-25* | 1.33 | 0.54 |
| IV-8G18 | *npr-26* | 0.86 | -0.23 |
| IV-8M21 | *npr-32* | -0.04 | -2.68 |
| V-1G19 | *T22H9.1* | 1.81 | -0.04 |
| V-4P14 | *dmsr-12* | 0.71 | 0.30 |
| V-5A11 | *dmsr-14* | -1.25 | -1.04 |
| V-5A13 | *dmsr-13* | 0.85 | 0.14 |
| V-5C05 | *frpr-18* | 0.52 | 0.17 |
| V-5G08 | *srsx-24* | 0.07 | 0.39 |
| V-5L13 | *frpr-3* | 1.32 | 1.54 |
| V-6O06 | *frpr-6* | 2.03 | 1.83 |
| V-7B04 | *npr-25* | 1.44 | -0.07 |
| V-7D16 | *dmsr-1* | 0.74 | 0.35 |
| V-7D24 | *T11F9.1* | 1.71 | 2.18 |

| **Geneservice location** | **Target** | **Proportion reversing** | |
| --- | --- | --- | --- |
|  |  | **initial** | **final** |
| V-7G01 | *egl-3* | -0.02 | 5.99 |
|  |  | -0.29 | 7.24 |
|  |  | 2.70 | 5.76 |
| V-8N12 | *frpr-5* | -0.05 | -0.22 |
| V-8O10 | *frpr-15* | 0.34 | -0.97 |
| X-1K03 | *npr-19* | 0.43 | 0.10 |
| X-2E06 | *npr-16* | 0.58 | -2.83 |
| X-2F18 | *npr-1* | -1.13 | -2.35 |
| X-2N15 | *nmur-3* | -0.72 | 1.22 |
| X-3J02 | *npr-8* | -0.23 | -1.08 |
| X-4C24 | *npr-28* | 0.73 | 0.13 |
| X-4D19 | *nmur-1* | -0.02 | -0.68 |
| X-4F14 | *npr-18* | -0.71 | -2.76 |
| X-4M14 | *frpr-8* | -0.60 | 0.17 |
| X-4N13 | *npr-7* | 0.07 | -0.85 |
| X-5E05 | *npr-6* | 2.02 | 2.72 |
| X-5P24 | *npr-4* | 1.76 | 1.82 |
| X-6I21 | *npr-10* | 0.38 | 0.14 |
| X-6J14 | *sprr-2* | 0.43 | 3.07 |
| X-7C08 | *npr-33* | 1.14 | 0.63 |
| X-7H14 | *npr-24* | 0.74 | 0.55 |
| X-7J08 | *frpr-7* | -0.04 | -1.25 |
| X-7M11 | *F59D12.1* | 0.69 | 0.55 |
| X-7P13 | *npr-21* | 0.96 | 0.27 |
