# Supplementary material for: Habituation as an adaptive shift in response strategy mediated by neuropeptides
Source: NPJ Sci Learn. 2017 Aug 18;2:9. doi: 10.1038/s41539-017-0011-8 (PMC6161508; doi:10.1038/s41539-017-0011-8)
Supplement: Supplementary file 2 — Supplementary Table 2 [file 41539_2017_11_MOESM2_ESM.docx]

Table S2. Cre promoters with full (++), partial (+), and no (-) rescuing capability when co-injected with floxed *pdfr-1* cDNA.

|  | **Habituation metric** | |  |
| --- | --- | --- | --- |
| **Cre promoter** | **latency** | **duration** | **Expression pattern** |
| *tag-168* | + + | - | pan-neuronal |
| *npr-1* | + | - | ~20 neuron classes – mostly sensory |
| *eat-4* | + | - | ~40 neuron classes – mostly sensory |
| *glr-1* | - | - | ~10 neuron classes – mostly interneurons |
| *gcy-36* | - | - | 3 neuron classes – sensory |
| *ocr-4* | - | - | 1 neuron class – sensory |
| *myo-3* | + | + | muscle |

Expression pattern references: *tag-168* (Ishihara & Katsura, unpublished), *npr-1*^1^, *eat-4*^2^, *glr-1*^3,4^, *gcy-36*^5^, *ocr-4*^6^, and *myo-3*^7^.-

References

1. Coates, J. C. & de Bono, M. Antagonistic pathways in neurons exposed to body fluid regulate social feeding in Caenorhabditis elegans. *Nature* **419,** 925–929 (2002).

2. Serrano-Saiz, E. *et al.* Modular control of glutamatergic neuronal identity in C. elegans by distinct homeodomain proteins. *Cell* **155,** 659–673 (2013).

3. Maricq, A. V., Peckol, E., Driscoll, M. & Bargmann, C. I. Mechanosensory signalling in C. elegans mediated by the GLR-1 glutamate receptor. *Nature* **378,** 78–81 (1995).

4. Hart, A. C., Sims, S. & Kaplan, J. M. Synaptic code for sensory modalities revealed by C. elegans GLR-1 glutamate receptor. *Nature* **378,** 82–85 (1995).

5. Cheung, B. H. H., Arellano-Carbajal, F., Rybicki, I. & de Bono, M. Soluble guanylate cyclases act in neurons exposed to the body fluid to promote C. elegans aggregation behavior. *Curr. Biol. CB* **14,** 1105–1111 (2004).

6. Tobin, D. M. *et al.* Combinatorial expression of TRPV channel proteins defines their sensory functions and subcellular localization in C. elegans neurons. *Neuron* **35,** 307–318 (2002).

7. Miller, D. M., Ortiz, I., Berliner, G. C. & Epstein, H. F. Differential localization of two myosins within nematode thick filaments. *Cell* **34,** 477–490 (1983).
