# Supplementary figures and images for: Habituation as an adaptive shift in response strategy mediated by neuropeptides
Source: NPJ Sci Learn. 2017 Aug 18;2:9. doi: 10.1038/s41539-017-0011-8 (PMC6161508; doi:10.1038/s41539-017-0011-8)

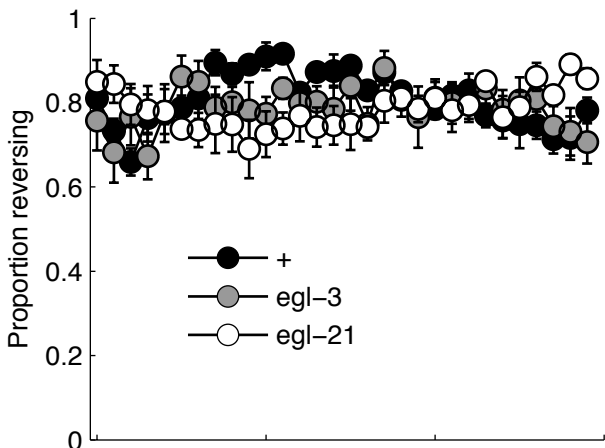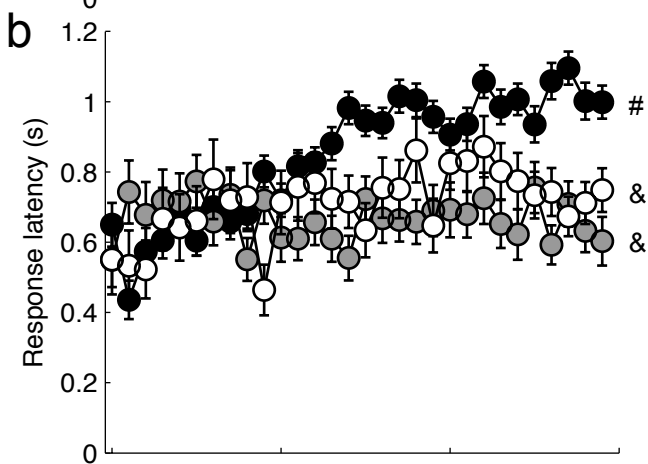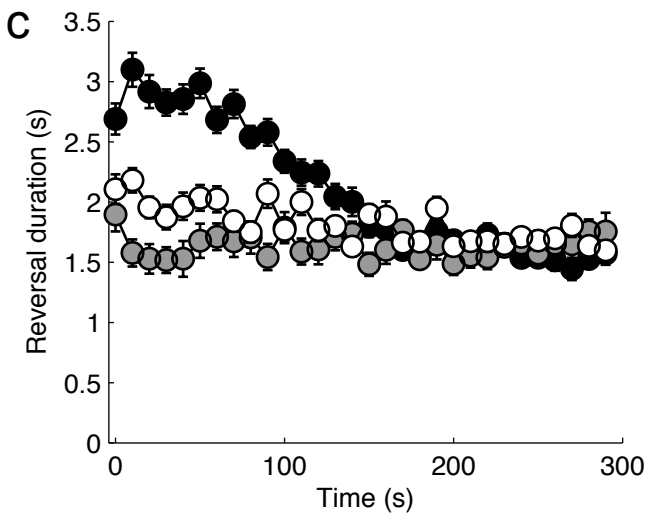

Supplement: Supplementary file 3 — Supplementary Figure 1 [file 41539_2017_11_MOESM3_ESM.pdf]

**a**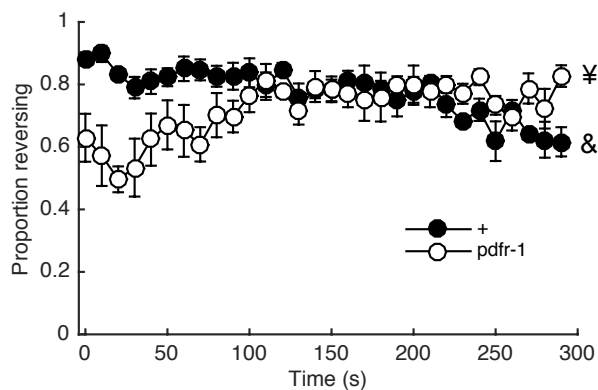**b**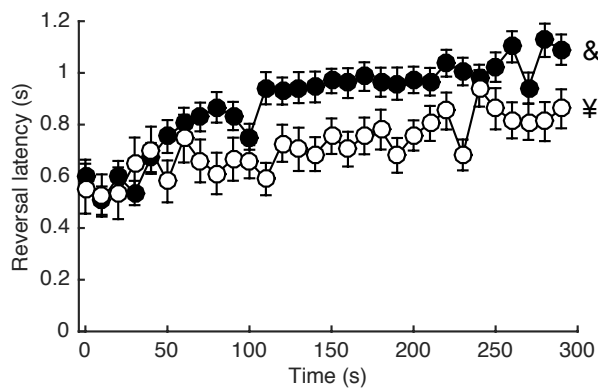**c**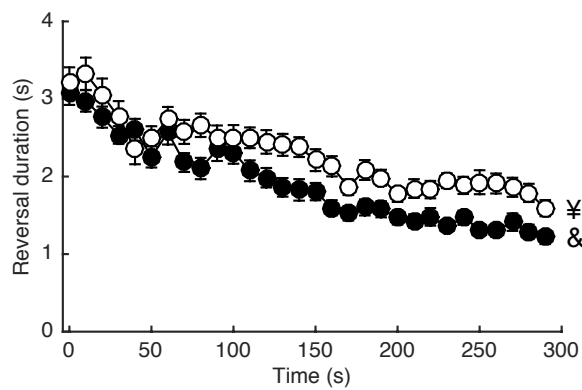**d**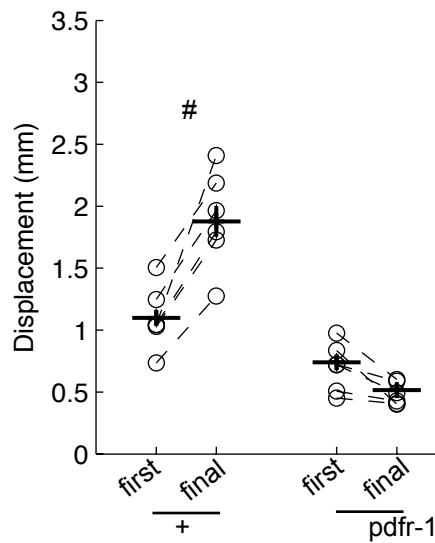

Supplement: Supplementary file 4 — Supplementary Figure 2 [file 41539_2017_11_MOESM4_ESM.pdf]

**a**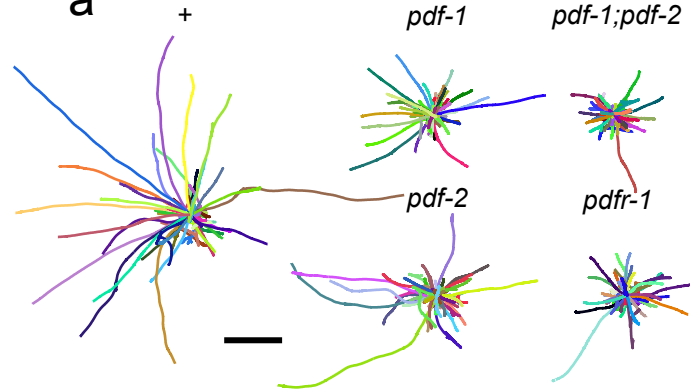**b**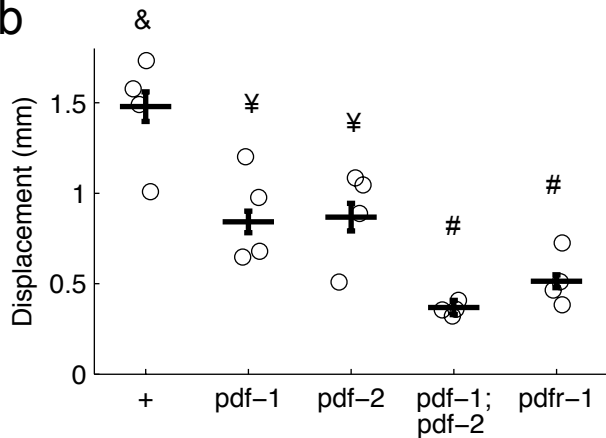

Supplement: Supplementary file 5 — Supplementary Figure 3 [file 41539_2017_11_MOESM5_ESM.pdf]
